# How and Why Do Smokers Start Using E-Cigarettes? Qualitative Study of Vapers in London, UK

**DOI:** 10.3390/ijerph13070661

**Published:** 2016-06-30

**Authors:** Elle Wadsworth, Joanne Neale, Ann McNeill, Sara C. Hitchman

**Affiliations:** 1 National Addiction Centre, King’s College London, 4 Windsor Walk, London SE5 8BB, UK; joanne.neale@kcl.ac.uk (J.N.); ann.mcneill@kcl.ac.uk (A.M.); sara.hitchman@kcl.ac.uk (S.C.H.); 2 UK Centre for Tobacco and Alcohol Studies, Nottingham NG5 1PB, UK

**Keywords:** electronic cigarettes, smoking cessation, nicotine, cigarettes, COM-B model, behaviour change

## Abstract

The aims of the study were to (1) describe how and why smokers start to vape and what products they use; (2) relate findings to the COM-B theory of behaviour change (three conditions are necessary for behaviour change (B): capability (C), opportunity (O), and motivation (M)); and (3) to consider implications for e-cigarette policy research. Semi-structured interviews (*n* = 30) were conducted in London, UK, with smokers or ex-smokers who were currently using or had used e-cigarettes. E-cigarette initiation (behaviour) was facilitated by: capability (physical capability to use an e-cigarette and psychological capability to understand that using e-cigarettes was less harmful than smoking); opportunity (physical opportunity to access e-cigarettes in shops, at a lower cost than cigarettes, and to vape in “smoke-free” environments, as well as social opportunity to vape with friends and family); and motivation (automatic motivation including curiosity, and reflective motivation, including self-conscious decision-making processes related to perceived health benefits). The application of the COM-B model identified multiple factors that may lead to e-cigarette initiation, including those that could be influenced by policy, such as price relative to cigarettes and use in smoke-free environments. The effects of these policies on initiation should be further investigated along with the possible moderating/mediating effects of social support.

## 1. Introduction

E-cigarettes are electronic devices that use a battery powered heating element to disperse liquid, usually a solution of glycerine and/or propylene glycol containing nicotine, into an aerosol that can be inhaled [1]. E-cigarettes can be classified into three main types: first-generation cigarette-like devices that are disposable or rechargeable with replaceable cartridges (cigalikes); second generation “tank” models that are designed to be refilled with liquid by the user (tanks) [2]; and third generation models that contain a higher capacity battery and are easily modified (mods). However, this categorisation continues to evolve. Since e-cigarettes do not burn tobacco and do not create smoke or rely on combustion, evidence indicates that they are a less harmful form of nicotine delivery than ordinary cigarettes, although the long-term effects are not yet known [1,2]. In England, e-cigarettes are now the most common quit aid used by smokers [3]. A Cochrane review found that e-cigarettes containing nicotine were better at helping smokers to stop smoking compared with placebo e-cigarettes [4].

Studies from the U.S. have shown that smokers initiate e-cigarette use after learning about them from advertising (including on the Internet) and through family and friends [5,6,7]. Others reported motivation for initiation to include curiosity, trying to quit or reduce smoking, the ability to vape in places where smoking is banned, alleviation of nicotine cravings, and health benefits and cost savings relative to cigarettes [7,8,9]. A limited number of qualitative studies have been carried out on adult e-cigarette users, with the majority of these conducted in the U.S., and with most only including current vapers. A common theme in all the studies is that relatively minimal and ambiguous health and product information deters e-cigarette use [10,11,12]. Similarities between cigarette smoking and vaping have also been found to facilitate switching [13], although some preferred flavours that were very different from cigarette smoke [14]. It is important to note, however, that both similarities and difference between cigarette smoking and vaping can facilitate switching to a similar extent in first time users [15].

To date, there has been no qualitative study using individual interviews of adult current and former vapers to understand the reasons for initiation. Additionally, no studies have used a theoretical framework to understand the factors the might lead smokers to initiate vaping. This paper, therefore, has the following three aims: (i) to describe how and why smokers start to vape, and what products they choose to initiate; (ii) to relate the findings to an established theory of behaviour change, the COM-B model [16]; and (iii) to consider the implications of the findings for policy research.

### The COM-B Model

The COM-B model has been applied to smoking behaviours [16], but has not yet been applied to vaping. It is based on the assumption that a person needs the capability (C) to execute any given behaviour (B); the opportunity (O) to take part in the behaviour; and the motivation (M) to engage in the behaviour over other competing behaviours. Moreover, in order for any given behaviour to change, these three conditions must be met [17]. Relating data on how and why smokers start to vape to the six core components of the COM-B model (i) physical capability; (ii) psychological capability; (iii) physical opportunity; (iv) social opportunity; (v) automatic motivation; and (vi) reflective motivation) should, therefore, provide useful insights into how the initiation of e-cigarettes might be better facilitated (for smokers for harm reduction) and prevented (for non-smokers) [18].

## 2. Materials and Methods

Analyses are based on data generated as part of a broader study looking at e-cigarette trajectories of use. The study received ethical approval from a university ethics committee (PNM/13/14-150) and involved semi-structured interviews (conducted between June and September 2014) with 30 current and ex-smokers who were using or who had used e-cigarettes at least weekly for a month in the past year. Participants included 13 males and 17 females; age range: 18–60 years. Further details relating to their smoking and e-cigarette (vaping) status are reported in Table 1 and their frequency of use in Table 2. Although all participants were recruited from, and lived in, London at the time of interview, they had diverse ethnic backgrounds (White British, White Irish, Other White, Mixed British, Other Asian British, and Black British) and nationalities (American, Australian, British, Irish, Italian, Lithuanian, Polish, Spanish, and Other Eastern European).

Participants were recruited via Gumtree, a UK website for free classified advertisements (*n* = 14), a university mailbase of research volunteers (*n* = 13), word of mouth (*n* = 2), and posters in local newsagents (*n* = 1). Our study aimed to recruit 30 individuals (maximum variation sampling) as the research team considered this to be both feasible and sufficient to generate new insights into e-cigarette trajectories of use. Interested individuals were invited to contact one of the study researchers by phone or email for further details. Those making contact were assured that all the information they provided would be kept confidential to the research team. They were also told that their participation was voluntary and they could remove themselves from the study at any time. After hearing more about the research, those who were still interested and eligible were offered an interview time of their choice and sent an information package, which contained written details about the study and a consent form.

Interviews took place in a private room at the university campus, were conducted in English, lasted approximately 50 min, and were audio recorded. Members of the research team developed a topic guide based on their knowledge of the existing literature, and gaps in policy and practice, and it comprised open questions with prompts and probes. The interview followed the topic guide that covered: smoking history; use of other nicotine containing products; views and experiences of e-cigarettes; cost of e-cigarettes; and e-cigarette marketing and regulations (Appendix A). On completion of the interview, participants were given £20 in compensation for their time, including travel expenses. All interviews were then transcribed verbatim, 11 interviews by the researchers and 19 by a professional transcription company. Transcriptions were imported into the qualitative software programme MaxQDA (v11) for systematic coding. MaxQDA is a product of VERBI GnbH, Berlin, Germany, and is an internationally recognized, field-tested, and journal-proven software package designed to facilitate the analysis of qualitative data. The coding frame developed comprised a mixture of deductive codes (based on the topic guide) and inductive codes (based on topics that emerged during the interviews).

One member of the research team coded the data, following discussion with the other team members, and the analyses were undertaken iteratively by team members, collectively. Coded data relating to how and why participants started to vape (deductive code labelled “initiation”) were exported from MaxQDA into a Microsoft Word (Microsoft Corporation, Redmond, WA, USA) document and analysed inductively line-by-line via iterative categorisation [19]. Iterative categorisation was chosen as it offered a rigorous and transparent approach to the analyses of textual data, whilst adhering to criteria of trustworthiness [20]. Themes emerging from the data were then mapped onto the six core COM-B components (physical capability; psychological capability; physical opportunity; social opportunity; automatic motivation; reflective motivation). Where themes potentially related to more than one COM-B component, members of the research team discussed the options and came to a joint decision on the best descriptor. In reporting the findings, all participants have been given a pseudonym to protect their anonymity.

## 3. Results

### 3.1. Physical Capability: Having the Physical Skills to Initiate E-Cigarette Use

Participants all indicated that they had had the physical capability/motor skills to enact the behaviour of vaping for the first time. Indeed, nobody stated that they had been unable to make the product work initially. Most participants initiated with a cigalike product, which were bought ready to use and easier than tank or modular devices, as most participants found these types to be ‘bulky’ and “scary”. Some individuals explained that they had observed their friends or family vaping cigalikes and then, because use was simple, had been able to replicate the action successfully.

*“It was hard to tell if, you know, you wanted the equivalent of one cigarette’s worth of nicotine, (yeah) it was hard to gauge how much of that you had to take in, so I wasn’t sure if it was more concentrated or not. Obviously, you can easily inhale, you know, and I think you usually get like a flash of light on the end.”* 

*(Yusef, current smoker and ex-vaper, aged 18–24 years)*

### 3.2. Psychological Capability: Having the Knowledge and Capacity to Initiate E-Cigarette Use

Overall, most participants reported that (1) they should quit smoking because it is harmful to their health; (2) recognised that e-cigarettes were comparatively better for them than cigarettes; (3) that they were a potentially useful smoking cessation aid; and (4) an alternative source of nicotine.

*“I almost use the e-cigarette to relieve myself from the dirt I’m putting into my system, to give myself a break, almost. So that way I’m still getting my nicotine… The problem with cigarettes, obviously, is that you see it’s not only the nicotine, it’s the other stuff and I’m very aware of that.”* 

*(Fraser, current smoker and ex-vaper, aged 35–49 years)*

However, a small number of participants acknowledged that they were not very knowledgeable about e-cigarettes, either explaining that they had heard conflicting messages or referring to the current lack of evidence on their safety and effectiveness:

*“One of the biggest negatives is the sort of constant, um, uneducated debate about it… even almost to the point of… I don’t actually know anything about them. I just use them and then you get, like, there’s a newspaper article or something (reporting that) these things are killing our kids.”* 

*(Connor, ex-smoker and current vaper, aged 18–24 years)*

Only a few participants used the internet to search for information about e-cigarettes prior to initiation. They described how they acquired the knowledge to choose an e-cigarette that might most effectively help them quit by searching for, and looking at, vaping and vaping products online. Several said that they had used social media, such as internet fora or Reddit, to increase their knowledge of product types before initiating. Thus, it seemed that, in addition to having the knowledge that e-cigarettes could be used to quit, some participants had more in-depth knowledge about e-cigarette product types, and knew to buy online or to go to specialty vape shops (see physical opportunity).

### 3.3. Physical Opportunity: Environmental Factors that Enable or Prompt Initiation of E-Cigarette Use

Most participants reported that cigalike e-cigarettes were readily available from shops (supermarkets, newsagents, and general stores, etc.) and this had facilitated their first use. For the most part, individuals did not need to go to specialist e-cigarette shops to make their initial purchases; but those that did purchased tank e-cigarettes. Some individuals said that it was the prominent position of an e-cigarette display at the shop counter that had persuaded them to buy their first product. A few participants who were already curious explained that the salesperson inside the shop had prompted them to buy their first e-cigarette:

*“He (shop assistant) was just, “Have you smoked or do you smoke?” and I was like, “Yeah” and (he) was like, “Try this”. He didn’t have to do much. I was curious about the product.”* 

*(Ethan, current smoker and current vaper, aged 18–24)*

According to a small number of participants, the increased presence of e-cigarettes both in shops and online had opened their eyes to the possibility of vaping. The availability of low cost cigalike e-cigarettes had facilitated first use because they were cheap enough for experimentation without financial commitment:

*“The local pound shop started selling like, er, disposable e-cigarettes with the promise that buying one was equivalent to like a pack of twenty. So £1* versus *£6, £7, £8. So you know, it seemed like a good deal.”* 

*(Yusef, current smoker and ex-vaper, aged 18–24)*

A small number of individuals also reported that having the opportunity to vape in public places or private homes had prompted them to initiate e-cigarette use. For example, one woman explained how she had starting using an e-cigarette because she could use it inside the pub without having to go outside. In addition, one man reported that he had first used an e-cigarette in the home of a friend because he had not wanted to go outside to smoke when visiting. Overall, the opportunity of vaping indoors seemed attractive to participants, although this was sometimes accompanied by uncertainty about the social acceptability; indeed one participant described searching online for information on vaping etiquette because they were unsure of the appropriateness of vaping in public places.

### 3.4. Social Opportunity: Social Factors that Enable or Prompt the Initiation of E-Cigarette Use

The majority of participants reported that they had heard about e-cigarettes from family or friends who were already vaping or who had vaped in the past. These individuals had often promoted the use of e-cigarettes to, or purchased products for, the participants because they wanted them to stop smoking. For example, Holly had first started vaping after her friend had physically gone to a specialist shop and bought her a tank e-cigarette:

*“I think it was my friend [who] encouraged it… She went and got it for me. She said, “I’ll go to the shop and get you one, give me the money”, and then she went and got it. She encouraged me…”* 

*(Holly, current smoker and current vaper, aged 25–34 years)*

Furthermore, one participant had no intention of quitting smoking but the persistence of their partner convinced them to quit and start vaping, as a compromise in the relationship. Sometimes, however, the encouragement of friends and relatives had inadvertently influenced participants*’* intentions and decisions about vaping negatively. This had occurred either because participants had felt too much pressure or because family or friends had promoted aspects of e-cigarette use that did not interest the participant; for example, the brother of one participant had advocated e-cigarettes on the grounds that they could be used indoors, but smoking outdoors did not concern the participant.

Participants also sometimes reported that the first e-cigarette they had used had actually belonged to another person, usually a friend or a colleague. In this situation, the participant either reported that they had been in an indoor environment, such as a pub, and had not wanted to go outside to smoke or that they were with a vaper who had spontaneously offered them their e-cigarette to try. A few individuals also clarified that they had experimented with e-cigarettes with friends or had planned to quit smoking using e-cigarettes with a friend:

*“When they tried those e-cigarettes a year or so ago, a mate goes to me, “It works”. So I was like, “I have to give it a try”. Then my brother, as well, my brother smokes, or used to smoke as well and he’s the one that got me on e-cigarettes as well recently.”* 

*(Liam, ex-smoker, ex-vaper, 25–34)*

Lastly, a few participants explained how seeing strangers vaping had initiated their interest. For example, one man said that an increase in the visibility of e-cigarettes in his surroundings in Italy had encouraged him to try a product.

### 3.5. Automatic Motivation: Feelings and Impulses that Affect the Initiation of E-Cigarette Use

Participants often spoke about “curiosity”, or their “desire” to try an e-cigarette, as being the main driver for initiation. Additionally, a few spoke of being attracted to e-cigarettes because they were “cool” or “fun”; for example, one participant described e-cigarettes as aesthetically “cool”, whereas another felt they were appealing because they were novel. In contrast, others explained how they had been reluctant to try e-cigarettes because they felt they were “silly” or “a fad”:

*“I remember thinking it was a fad, like the herbal cigarettes my Mum used to smoke. I just remember thinking they were silly, so I never really thought anything of it, and then I tried them and realised it was actually quite effective.”* 

*(Grace, ex-smoker and ex-vaper, aged 25–34)*

One individual also noted how it was the *‘*enjoyment*’* of smoking tobacco cigarettes—particularly the smell and taste—that had, for a long time, deterred her from starting to vape (before her boyfriend*’*s persistence and love of exercise triggered her to try e-cigarettes):

*“I love the smell of cigarettes. I love the way they taste, the disgusting taste that’s perfect, the smoke that burns. That’s something that I really enjoy... My boyfriend tried to convince me (to try an e-cigarette)… and I said… “I don’t want to do it. Just leave me alone so I can smoke my cigarettes”.”* 

*(Una, ex-smoker and current vaper, aged 25–34 years)*

### 3.6. Reflective Motivation: Self-Conscious Decision Making and Reasoning that Influence the Initiation of E-Cigarette Use

In addition to feelings and impulses, many participants gave considered reasons that motivated them to initiate vaping. For example, some reported that they had been happy to try e-cigarettes because they had wanted to improve their health:

*“If I’m gonna be an addict to an e-cigarette, it’s gotta be better. I mean, if all I’m gonna go do is swap my addiction over to an e-cigarette then, I think it’s worth doing.”* 

*(Holly, current smoker and current vaper, aged 25–34 years)*

Others explained that they had initiated vaping because they believed that e-cigarettes would alleviate their cravings and help them to stop smoking. In particular, some reflected on how the hand-to-mouth actions of smoking and vaping were very similar, with both satisfying a psychological need to put something in the mouth. A few individuals also explained that the *‘*tactile*’* similarities between an e-cigarette and a cigarette had motivated them to initiate vaping. These attributes made vaping more attractive to them than nicotine patches or gum:

*“Just chewing gum or putting a patch on, it’s not the same. You need to have your cigarette, like light up and everything… So… it’s either you have your normal cigarettes and like cut down or go for the e-cigarette. So obviously I decided to go for the e-cigarette.”* 

*(Belle, ex-smoker and ex-vaper, aged 18–24 years)*

Conversely, a number of participants reported that they were initially not motivated to try e-cigarettes because they had felt that they would not work or did not replicate the process of smoking. Still, others lacked motivation to vape initially because they were using e-cigarettes as a quit aid and felt that e-cigarettes replicated the hand-to-mouth action of a cigarette too closely and, therefore, did not break the psychological habit of smoking.

A summary of the COM-B model categories can be found in Table 3.

## 4. Discussion

Using the COM-B model [16], our study showed that behaviour (e-cigarette initiation) was facilitated by individuals having the capability (the physical skills, and the knowledge and capacity to initiate e-cigarette use); the opportunity (access to environments and social situations that enable or prompt initiation of e-cigarette use); and the motivation (feelings, impulses, and self-conscious decision-making that affect initiation of e-cigarette use). Conversely, e-cigarette initiation was prevented by lack of capability (inadequate evidence about the safety and effectiveness of e-cigarettes relative to smoking tobacco); lack of opportunity (uncertainty about the social acceptability of vaping (particularly in public places), and negative social pressure to vape (or family and friends promoting benefits of vaping that did not personally interest them); and the lack of motivation (arising from a belief that e-cigarettes were silly or a fad, deep enjoyment of smoking, a belief that e-cigarettes did not work or could not replace smoking, and concern that the hand-to-mouth action of using an e-cigarette was too similar to smoking to break the habit).

Aspects of our findings are consistent with previous research. E-cigarette initiation was facilitated by the influence of family or friends [6,7], the ability to vape in places where smoking is not allowed [9,21], the lower cost of e-cigarettes compared to tobacco cigarettes [8,9], the desire to reduce cigarette consumption or quit smoking [7,21,22], and curiosity [7,9,23]. However, application of the COM-B model also revealed newer insights:

In terms of capability, we found that few individuals performed any prior research on how to use an e-cigarette or what type would be most effective to help them quit; most purchased on impulse. Cigalikes were the most common type of product first used, reflecting their greater availability in general shops. There was also evidence that those who started vaping thought that, despite safety concerns, e-cigarettes were less harmful than tobacco smoking; in other words, they understood and made decisions based on the concept of relative harm. However, some participants were confused about the safety of e-cigarettes. There was a clear desire for information about e-cigarettes, including research evidence on what is known about their harmfulness compared to smoking [24]. How to communicate the relative harmfulness information about e-cigarettes and smoking to smokers for harm reduction, and the best sources of information (e.g., health warnings, media campaigns), warrants further research.

In terms of opportunity, there seemed to be a strong interaction between social and physical opportunities to vape, such that family and friends often facilitated vaping, but in specific places, such as pubs, where smoking was not allowed. Allowing vaping where smoking is not allowed enabled some smokers to take advantage of the environmental opportunity to try e-cigarettes (often enhanced through a social interaction). Such policies are, however, not uncontroversial. Concerns have been expressed that allowing e-cigarette use in public places may encourage initiation among some youth and young adults, thus, further research is needed in these areas [25]. Families and friends also provided social support to those using e-cigarettes to quit smoking, and for some participants it was primarily the social support that helped them make the transition. Future policies that encourage the switch to e-cigarettes from cigarettes should consider and incorporate the possible moderating/mediating effects of social support. Advertising at the point of sale and availability of e-cigarettes encouraged smokers to try e-cigarettes. Retailers also appeared to play a role in initiation by suggesting e-cigarette trial, and supporting retailers in this role with education about e-cigarettes might support them in their efforts to disinvest from tobacco sales [26]. Our findings additionally suggest that research into policies related to e-cigarettes and the interaction with tobacco control policies is necessary. For example, the lower cost of e-cigarettes relative to smoking in the UK seems to provide smokers with the opportunity to try e-cigarettes. Policy research should examine how the costs of initiating vaping vs. continuing to smoke may influence uptake among smokers.

In terms of motivation, the hand-to-mouth action of e-cigarettes appeared to encourage some smokers to vape as it resembled smoking but deterred others as it was too similar. Additionally, similar to studies showing that enjoyment of smoking predicts quit attempts [27], deep enjoyment of smoking was also a barrier to e-cigarette initiation. Opinions varied on all products available, for example tanks were appealing to some whilst a deterrent for others. One size will not fit all and, therefore, keeping a wide selection of products is likely to be beneficial in capturing most smokers looking to quit.

### Limitations and Strengths

This study used the qualitative study design of self-selection, which creates a non-representative sample of e-cigarette users. In addition, this study focused on smokers and ex-smokers recruited and interviewed in London. As such, our findings cannot be generalized empirically to other populations or settings, but consistency with some other research suggests our findings are not atypical. The COM-B model enabled us to identify factors that both facilitated and impeded the initiation of vaping amongst smokers. However, we experienced some difficulty in isolating several components of the model. For example, understanding that smoking is bad for health (capability) is difficult to separate from the resultant desire to quit (motivation). In addition, it is possible that alternative factors that enable or prompt initiation of e-cigarette use, such as additional environmental or social factors, were not considered by participants. We experienced difficulty in separating the three components into their respective subcategories (physical capability; psychological capability; social opportunity; physical opportunity; automatic motivation; and reflective motivation). These are the first qualitative data on e-cigarette initiation to be applied to the COM-B model, therefore, a strength of the study is that it provides a new way that qualitative data could be analysed for informing intervention design and policy research. The diversity of individuals interviewed was a key strength of the study. Participants reported a wide age range (18–60 years), many ethnic backgrounds and nationalities, and varied patterns of e-cigarette usage. The study was exploratory and numbers were too small for meaningful subgroup analyses, however, the sample size of 30 was small enough not to require excess time and money, yet large enough to collect rich and in-depth data on a new phenomenon. Additionally, most research to-date has tended to rely on quantitative data and available qualitative studies (mainly U.S) have largely used focus groups or not focused on initiation. Future research could explore past initiation to what factors influence vapers to continue or stop use, and what policies influence the transition between cigarettes and e-cigarettes.

## 5. Conclusions

To conclude, we return to our original three aims. First, a range of factors influenced e-cigarette initiation amongst smokers. Most importantly, these included an understanding of the concept of harm reduction, easy access to cheap cigalikes in shops, particularly where sales staff talked to customers about them, encouragement from social network members, ability to vape in public places, and belief in their effectiveness; second, the COM-B model of behaviour change provided a useful heuristic for exploring e-cigarette initiation, and enabled us to identify a number of factors not previously reported in the literature; third, a co-ordinated approach to policy research on e-cigarettes could encompass tailored information for smokers, promotion at point of sale, a sensible price/taxation policy relative to smoking, and a strategy and clear guidance on where vaping is permitted and permissive vaping policies where appropriate. Nonetheless, it is also important to recognise that e-cigarettes are only one of a range of quit smoking aids [28,29] and any information or guidance should reference other smoking cessation and harm reduction support available.

## Figures and Tables

**Table 1:** Participants’ key demographic characteristics by smoking and vaping status at the time of the interview.

Demographics	Ex-Smoker Ex-Vaper	Ex-Smoker Current Vaper	Current Smoker Ex-Vaper	Current Smoker Current Vaper	Total
**Age (Years)**	*n* = 5	*n*= 7	*n* = 9	*n* = 9	***n* = 30**
**18–24**	2	1	3	3	**9**
**25–34**	2	2	2	4	**10**
**35–49**	1	3	3	1	**8**
**50–59**	0	0	1	1	**2**
**60+**	0	1	0	0	**1**
**Gender**					
**Female**	3	5	4	5	**17**
**Male**	2	2	5	4	**13**

Smokers were defined as those who have smoked in the last 30 days; ex-smokers were defined as those who had, at minimum, quit smoking in the past year; current vapers were defined as those who have vaped at least weekly in the past month; and ex-vapers were defined as those who had not vaped in the past month.

**Table 2:** Participants’ smoking and vaping status at time of interview.

Participants’ Smoking and Vaping Use at Time of Interview	Daily	Weekly	Monthly	Total No. of Participants
**Dual users**				
Smoking status	5	4	0	**9**
Vaping status	3	5	1	
**Smokers**				
Smoking status	9	0	0	**9**
**Vapers**				
Vaping status	**6**	**1**	**0**	**7**

**Table 3:** Categories of the COM-B model and examples of narratives from participants.

Themes	Physical Capability	Psychological Capability	Physical Opportunity	Social Opportunity	Automatic Motivation	Reflective Motivation
Narratives	“it was hard to tell if, you know, you wanted the equivalent of one cigarette’s worth of nicotine, (yeah) it was hard to gauge how much of that you had to take in, so I wasn’t sure if it was more concentrated or not. Obviously, you can easily inhale, you know, and I think you usually get like a flash of light on the end.” (Yusef, current smoker and ex-vaper, aged 18–24 years).	“I almost use the e-cigarette to relieve myself from the dirt I’m putting into my system, to give myself a break, almost. So that way I’m still getting my nicotine… The problem with cigarettes, obviously, is that you see it’s not only the nicotine, it’s the other stuff and I’m very aware of that.” (Fraser, current smoker and ex-vaper, aged 35–49 years).	“He (shop assistant) was just, “Have you smoked or do you smoke?” and I was like, “Yeah”and (he) was like, ”Try this”. He didn’t have to do much. I was curious about the product.” (Ethan, current smoker and current vaper, aged 18–24).	“I think it was my friend (who) encouraged it… She went and got it for me. She said, “I’ll go to the shop and get you one, give me the money”, and then she went and got it. She encouraged me…” (Holly, current smoker and current vaper, aged 25–34 years).	“I remember thinking it was a fad, like the herbal cigarettes my Mum used to smoke. I just remember thinking they were silly, so I never really thought anything of it, and then I tried them and realised it was actually quite effective.” (Grace, ex-smoker and ex-vaper, aged 25–34).	“If I’m gonna be an addict to an e-cigarette, it’s gotta be better. I mean, if all I’m gonna go do is swap my addiction over to an e-cigarette then, I think it’s worth doing.” (Holly, current smoker and current vaper, aged 25–34 years).
		“One of the biggest negatives is the sort of constant, um, uneducated debate about it… even almost to the point of… I don’t actually know anything about them. I just use them and then you get, like, there’s a newspaper article or something (reporting that) these things are killing our kids.” (Connor, ex-smoker and current vaper, aged 18–24 years).	“The local pound shop started selling like, er, disposable e-cigarettes with the promise that buying one was equivalent to like a pack of twenty. So £1 versus £6, £7, £8. So you know, it seemed like a good deal.” (Yusef, current smoker and ex-vaper, aged 18–24).	“When they tried those e-cigarettes a year or so ago, a mate goes to me, “It works”. So I was like, “I have to give it a try”. Then my brother, as well, my brother smokes, or used to smoke as well and he’s the one that got me on e-cigarettes as well recently.” (Liam, ex-smoker, ex-vaper, 25–34).	“I love the smell of cigarettes. I love the way they taste, the disgusting taste that’s perfect, the smoke that burns. That’s something that I really enjoy... My boyfriend tried to convince me (to try an e-cigarette)… and I said… “I don’t want to do it. Just leave me alone so I can smoke my cigarettes”.” (Una, ex-smoker and current vaper, aged 25–34 years).	“Just chewing gum or putting a patch on, it’s not the same. You need to have your cigarette, like light up and everything… So… it’s either you have your normal cigarettes and like cut down or go for the e-cigarette. So obviously I decided to go for the e-cigarette.” (Belle, ex-smoker and ex-vaper, aged 18–24 years).

## Appendix A

Condensed Interview Guide: Ex-smokers

(1) Smoking history
  - Smoking initiation/reasons for starting and continuing to smoke.
  - How much did you used to smoke?
  - Changes in smoking patterns over the years (attempts to quit/cutting down).
  - Reasons/motivations for quitting smoking (help with quitting plan?).
(2) Use of other nicotine containing products
  - Use of nicotine gum, patch, lozenges, tablets nasal or mouth spray, inhaler, oral strips or film. (Medications: Bupropion (Wellbutrin/Zyban) or Varenicline (Champix).
(3) Experience of E-cigarettes
  - E-cigarette initiation.
  - E-cigarettes used for current successful attempt?
  - Advice about products.
  - Reasons for current e-cigarette use/reasons for stopping e-cigarette use.
  - Current use and changes in e-cigarette use patterns (historical account of each product/ device used-transition over time/appearance or description of products/nicotine levels/flavouring/ frequency of use).
  - Overall satisfaction with each product/device.
  - Ultimate goal in e-cigarette product use, future plans.
  - Description of how product(s) changed their daily routine (comparable to smoking).
  - When and where they use.
  - Social aspects of use (also used by peer group/social groups/family/colleagues/etc.).
  - Reactions of others (variation in time, places, people, type of device, etc.).
  - General feelings about e-cigarettes (positive and negative).
(4) Cost
  - Where or how do they acquire their e-cigarettes.
  - How much money have they paid for a product.
  - History of prices.
  - How much are they prepared to pay.
  - Cost savings from regular tobacco.
(5) E-cigarette management and regulations
  - Health and safety concerns.
  - Views on push to regulate e-cigarettes as medicinal product under the Medicines and Healthcare Products Regulatory Agency.
  - Views on whether they should be used as part of a structured quitting plan.
  - What do you think e-cigarettes should be used for? (Harm reduction tool/ smoking cessation/ support for smoking withdrawal, etc.).
  - Views on marketing and advertising of e-cigarettes.
  - Check if the interviewee wants to add anything else or ask any questions.

Condensed Interview Guide: Current Smokers

(1) Smoking history
  - Smoking initiation/reasons for starting and continuing to smoke.
  - How much do you smoke?
  - Changes in smoking patterns over the years (attempts to quit/cutting down).
  - Future interest in smoking cessation or not (if so, reasons/motivations for wanting to quit/help with quitting plan?).
(2) Use of other nicotine containing products
  - Use of nicotine gum, patch, lozenges, tablets nasal or mouth spray, inhaler, oral strips, or film. (Medications: Bupropion (Wellbutrin/Zyban) or Varenicline (Champix).
(3) Experience of E-cigarettes
  - E-cigarette initiation.
  - Advice about products.
  - Reasons for current e-cigarette use/Reasons for stopping e-cigarette use.
  - Current use and changes in e-cigarette use patterns (historical account of each product/device used-transition over time/appearance or description of products/nicotine levels/flavouring/frequency of use).
  - Overall satisfaction with each product/device.
  - Ultimate goal in e-cigarette product use, future plans.
  - Description of how product(s) changed their daily smoking routine, comparable to smoking.
  - When and where they use.
  - Social aspects of use (also used by peer group/ social groups/family/colleagues/etc.).
  - Reactions of others (variation in time, places, people, type of device, etc.).
  - General feelings about E-cigarettes (positive and negative).
(4) Cost
  - Where or how do they acquire their e-cigarettes.
  - How much money have they paid for a product.
  - History of prices.
  - How much are they prepared to pay.
  - Cost savings from regular tobacco.
(5) E-cigarette management and regulations
  - Health and safety concerns.
  - Views on push to regulate E-cigarettes as medicinal product under the Medicines and Healthcare Products Regulatory Agency (MHRA).
  - Views on whether they should be used as part of a structured quitting plan.
  - What do you think e-cigarettes should be used for? (Harm reduction tool/smoking cessation/support for smoking withdrawal).
  - Views on marketing and advertising of e-cigarettes.
  - Check if the interviewee wants to add anything else or ask any questions.

## References

[1] Goniewicz M.L., Lingas E.O., Hajek P. (2013). Patterns of electronic cigarette use and user beliefs about their safety and benefits: An internet survey. Drug Alcohol Rev..

[2] Farsalinos K.E., Polosa R. (2014). Safety evaluation and risk assessment of electronic cigarettes as tobacco cigarette substitutes: A systematic review. Ther. Adv. Drug Saf..

[3] West R., Beard E., Brown J. Trends in Electronic Cigarette Use in England. www.smokinginengland.info/latest-statistics.

[4] McRobbie H., Bullen C., Hartmann-Boyce J., Hajek P. (2014). Electronic cigarettes for smoking cessation and reduction. Cochrane Database Syst. Rev..

[5] Barbeau A.M., Burda J., Siegel M. (2013). Perceived efficacy of e-cigarettes versus nicotine replacement therapy among successful e-cigarette users: A qualitative approach. Addict. Sci. Clin. Pract..

[6] McQueen A., Tower S., Sumner W. (2011). Interviews with “vapers”: Implications for future research with electronic cigarettes. Nicotine Tob. Res..

[7] Pepper J.K., Ribisl K.M., Emery S.L., Brewer N.T. (2014). Reasons for starting and stopping electronic cigarette use. Int. J. Environ. Res. Public Health.

[8] Farsalinos K.E., Romagna G., Tsiapras D., Kyrzopoulos S., Voudris V. (2014). Characteristics, perceived side effects and benefits of electronic cigarette use: A worldwide survey of more than 19,000 consumers. Int. J. Environ. Res. Public Health.

[9] Kong G., Morean M.E., Cavallo D.A., Camenga D.R., Krishnan-Sarin S. (2014). Reasons for electronic cigarette experimentation and discontinuation among adolescents and young adults. Nicotine Tob. Res..

[10] Coleman B.N., Johnson S.E., Tessman G.K., Tworek C., Alexander J., Dickinson D.M., Rath J., Green K.M. (2015). “It’s not smoke. It’s not tar. It’s not 4000 chemicals. Case closed”: Exploring attitudes, beliefs, and perceived social norms of e-cigarette use among adult users. Drug Alcohol Depend..

[11] McDonald E.A., Ling P.M. (2015). One of several “toys” for smoking: Young adult experiences with electronic cigarettes in New York City. Tob. Control.

[12] Rooke C., Cunningham-Burley S., Amos A. (2015). Smokers’ and ex-smokers’ understanding of electronic cigarettes: A qualitative study. Tob. Control.

[13] Pearson J.L., Smiley S.L., Rubin L.F., Anesetti-Rothermel A., Elmasry H., Davis M., DeAtley T., Harvey E., Kirchner T., Abrams D.B. (2016). The moment study: Protocol for a mixed method observational cohort study of the alternative nicotine delivery systems (ands) initiation process among adult cigarette smokers. BMJ Open.

[14] Simmons V.N., Quinn G.P., Harrell P.T., Meltzer L.R., Correa J.B., Unrod M., Brandon T.H. (2016). E-cigarette use in adults: A qualitative study of users’ perceptions and future use intentions. Addict. Res. Theory.

[15] Caponnetto P., Maglia M., Polosa R. (2015). Commentary on Dawkins et al. Electronic cigarettes—from smoking cessation to smoking sensation and back. Addiction.

[16] Michie S., van Stralen M.M., West R. (2011). The behaviour change wheel: A new method for characterising and designing behaviour change interventions. Implement. Sci..

[17] West R., Brown J. (2013). Theory of Addiction.

[18] NICE (2013). Smoking: Harm Reduction.

[19] Neale J. (2016). Iterative categorization (ic): A systematic technique for analysing qualitative data. Addiction.

[20] Shenton A.K. (2004). Strategies for ensuring trustworthiness in qualitative research projects. Educ. Inform..

[21] Dockrell M., Morison R., Bauld L., McNeill A. (2013). E-cigarettes: Prevalence and attitudes in Great Britain. Nicotine Tob. Res..

[22] Action on Smoking and Health (ASH) Use of Electronic Cigarettes (Vapourisers) Among Adults in Great Britain. http://www.ash.org.uk/files/documents/ASH_891.pdf.

[23] Schmidt L., Reidmohr A., Harwell T.S., Helgerson S.D. (2014). Prevalence and reasons for initiating use of electronic cigarettes among adults in Montana, 2013. Prev. Chronic Dis..

[24] Brose L.S., Hitchman S.C., Brown J., West R., McNeill A. (2015). Is the use of electronic cigarettes while smoking associated with smoking cessation attempts, cessation and reduced cigarette consumption? A survey with a 1-year follow-up. Addiction.

[25] Choi K., Fabian L., Mottey N., Corbett A., Forster J. (2012). Young adults’ favorable perceptions of snus, dissolvable tobacco products, and electronic cigarettes: Findings from a focus group study. Am. J. Public Health.

[26] Hitchman S.C., Calder R., Rooke C., McNeill A. (2016). Small retailers’ tobacco sales and profit margins in two disadvantaged areas of england. Aims Public Health.

[27] Fidler J.A., West R. (2011). Enjoyment of smoking and urges to smoke as predictors of attempts and success of attempts to stop smoking: A longitudinal study. Drug Alcohol Depend..

[28] Cahill K., Stevens S., Perera R., Lancaster T. (2013). Pharmacological interventions for smoking cessation: An overview and network meta-analysis. Cochrane Database Syst. Rev..

[29] Heydari G., Masjedi M., Ahmady A.E., Leischow S.J., Lando H.A., Shadmehr M.B., Fadaizadeh L. (2014). A comparative study on tobacco cessation methods: A quantitative systematic review. Int. J. Prev. Med..

